# Data on zinc chelates of glycine as a growth and survival enhancer in hybrid grouper and whiteleg shrimp

**DOI:** 10.1016/j.dib.2025.111309

**Published:** 2025-01-14

**Authors:** Sarmila Muthukrishnan, Donny Kai Hee Tey, Wen Chen Wee

**Affiliations:** a Department of Science and Technology Studies, Faculty of Science, University of Malaya, 50603 Kuala Lumpur, Malaysia; b Envitgro Pte Ltd, 10 Anson Road #33-07/08. International Plaza Singapore, 079903, Singapore

**Keywords:** Zinc chelates of glycine, Hybrid grouper, Whiteleg shrimp, Growth performance & survival

## Abstract

The dataset demonstrates the efficacy of Aqua Ectogon-284, a zinc chelates of glycine, as a dietary supplement to enhance growth and survival in hybrid grouper (*Epinephelus fuscoguttatus* ♀ × *E. lanceolatus* ♂) and whiteleg shrimp (*Penaeus vannamei*). Two concentrations (1g/kg feed and 2g/kg feed) were evaluated against a control group to identify an optimal dosage that maximizes growth and health outcomes. Both the species exhibited improved growth performance and survival rates with dietary supplementation. The hybrid grouper fed a 1g/kg diet achieved the highest final weight of 100.00 g, with significant increases in length and survival rates of 96.00%. Similarly, whiteleg shrimp demonstrated enhanced final weights and survival (94.67%) with the same treatment. These findings revealed that zinc chelate glycine increased the growth rate and reduced mortality in farmed species.

Specifications Table

**Table:** 

Subject	Agricultural science, Aquaculture
Specific subject area	Zinc chelates of glycine as a growth and survival enhancer
Type of data	Figures and tables
Data collection	The survival rate (%), final weight (g), final length (cm), weight gain (g) and specific growth rate (SGR, %) of the hybrid grouper and whiteleg shrimp were determined based on the following equations: Survival (%) = (final amount of animal /initial amount of animal) X 100% Weight Gain (g) = final weight (g) – initial weight (g) Final Length (cm) = final length (cm) – initial length (cm) Specific Growth rate (%/day) = $\frac{\ln(final\;weight\;(g))-\ln(initial\;weight\;(g))}{Feeding\;Period\;(days)}X\;100\%$
Data source location	Region: Kampung Sungai Sembilang, Selangor. Latitude and longitude for the feeding trials at experimental sites: 3.2075°N, 101.3045°E Data: Feb 2024 – April 2024
Data accessibility	Repository name: Sarmila Muthukrishnan; Wee Wen Chen & Donny Tey Kai Hee (Muthukrishnan, Sarmila; Wee, Wen Chen; Tey, Donny Kai Hee (2024), “Zinc chelates of glycine as a growth and survival enhancer in hybrid grouper and whiteleg shrimp”, Mendeley Data, V1, doi: 10.17632/8sc8n33tmh.1) Data identification number: doi: 10.17632/8sc8n33tmh.1 Direct URL to data: https://data.mendeley.com/datasets/8sc8n33tmh/1

## 1 Value of the Data

• The dataset documented enhanced growth performance of both the hybrid grouper and whiteleg shrimp which suggests that dietary supplementation with a zinc chelate glycine can promote better growth rates.
• Supplementation with 0.1% zinc chelate of glycine, which was found to be sufficient, effectively increased survival rates in both aquatic species.
• The use of zinc chelate of glycine aligns with the current trends toward more sustainable and environmentally friendly aquaculture practices while maintaining production efficiency.
• The findings underscore the role of dietary supplements, particularly chelated trace minerals like zinc, in enhancing stress tolerance and immune function. This is crucial for aquaculture species facing challenges from high-density rearing conditions that can lead to oxidative stress and weakened immune responses.

## 2 Background

Current trends in aquaculture focuses more on the sustainability, environmentally friendly, and the delivering of safe aquaculture product for consumers [1,5]. Among the various factors influencing fish welfare, dietary supplementation plays a major role in to cope with stress tolerance, health, and disease resistance [6]. Zinc (Zn), is one of the essential trace minerals for various biological functions, including cell structure development, enzymatic activity, immune system function, and protein and carbohydrate metabolism (Watanabe and Shuich, [8]; NRC, [4]). Serving as both a catalytic and structural cofactor for numerous proteins, adequate zinc intake is necessary for maintaining optimum growth and health resilience [3]. Due to their higher absorption and availability compared to inorganic sources, chelated trace minerals are gaining strong interest among feed manufacturers and animal producers, making them a popular choice for enhancing animal performance [2,7].

This study investigates the efficacy of a Zinc chelated glycine supplement, in enhancing growth and survival in hybrid grouper (*Epinephelus fuscoguttatus* ♀ × *E. lanceolatus* ♂) and whiteleg shrimp (*Penaeus vannamei*). The trials aimed to determine optimal concentrations of a Zinc chelated glycine supplement under commercial aquaculture conditions. By evaluating two concentrations, 0.1% and 0.2%, against a control group, we aim to determine an optimal dosage that enhances growth and health outcomes without adverse effects.

## 3 Data Description

Here, we provide the dataset of the growth performance and survival of the hybrid grouper and whiteleg shrimp fed with feed coated with zinc chelate of glycine. The raw data is available at https://data.mendeley.com/datasets/8sc8n33tmh/1. The raw data consists of:

1. Growth performance and survival of hybrid grouper and whiteleg shrimp
2. Environmental variables measured and raw data of hybrid grouper experiments.
3. Environmental variables measured and raw data of whiteleg shrimp experiments.

**Fig. 1:**
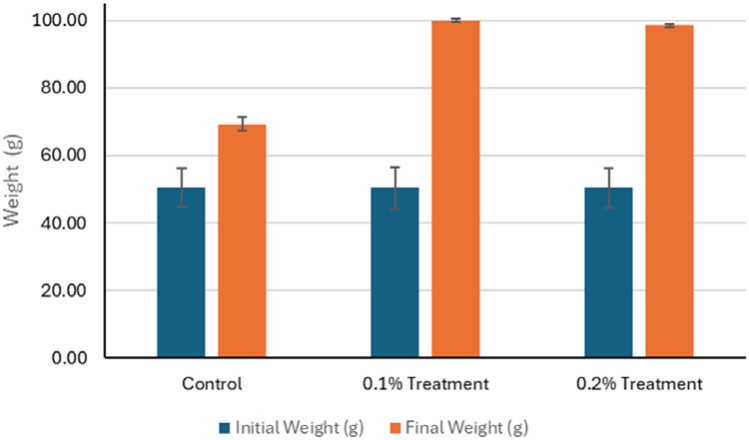
The initial weight (g) and final weight (g) of the hybrid groupers in control and treatment after 30 days of trials. The control group feed was prepared without the inclusion of zinc chelate of glycine. As for the treatment 0.1, feed was top-coated with zinc chelate of glycine at a concentration of 1 g/kg of feed and for the treatment 0.2, feed was top-coated with zinc chelate of glycine at a concentration of 2 g/kg of feed. Error bars represents the standard deviation of the replicates.

**Fig. 2:**
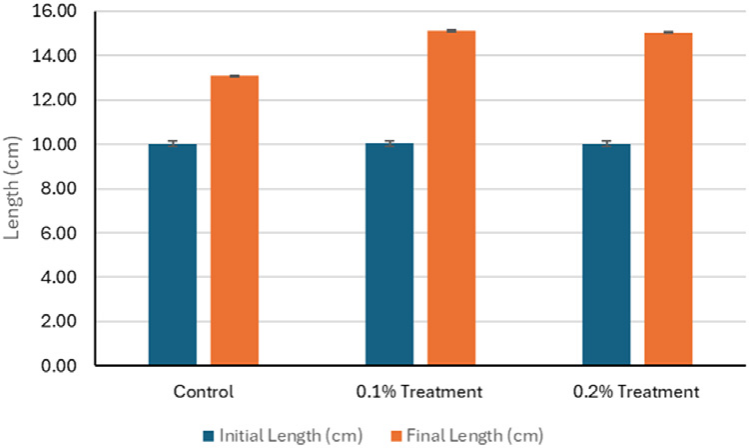
The initial length (cm) and final length (cm) of the hybrid groupers in control and treatment after 30 days of trials. The control group feed was prepared without the inclusion of zinc chelate of glycine. As for the treatment 0.1, feed was top-coated with zinc chelate of glycine at a concentration of 1 g/kg of feed and for the treatment 0.2, feed was top-coated with zinc chelate of glycine at a concentration of 2 g/kg of feed. Error bars represents the standard deviation of the replicates.

**Fig. 3:**
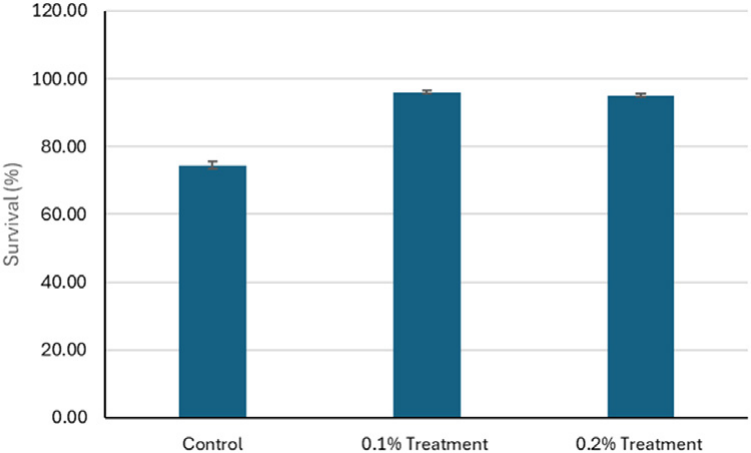
The survival (%) of the hybrid groupers in control and treatment after 30 days of trials. The control group feed was prepared without the inclusion of zinc chelate of glycine. As for the treatment 1 g/kg feed 0.1, feed was top-coated with zinc chelate of glycine at a concentration of 1 g/kg of feed and for the treatment 0.2, feed was top-coated with zinc chelate of glycine at a concentration of 2 g/kg of feed. Error bars represents the standard deviation of the replicates.

The effects of 1g/kg (0.1%) and 2g/kg (0.2%) feed coated with zinc chelate of glycine on growth and survival of hybrid grouper and whiteleg shrimp compared to the control groups were investigated. The initial weights of hybrid grouper were similar across groups, but final weights showed significant increase, with the 0.1% zinc chelate of glycine fed group achieving the highest weight at 100.00 g (Fig. 1). The final length of the hybrid grouper treated with zinc chelate of glycine also increased, reaching 15.12 cm and 15.05 cm, respectively, compared to 13.12 cm in the control (Fig. 2). Survival rates improved significantly (*P* < 0.05) with zinc chelate of glycine, with 96.00% survival rate reported for 0.1% of treatment and 95.00% of survival rate reported for 0.2% of treatment, compared to 74.50% in the control (Fig. 3). Weight gain (g) was markedly higher in the treatment groups (49.68 g/0.1% treatment and 48.16 g/0.2% treatment), leading to higher average daily gains (ADG) of 1.66 g/d and 1.61 g/d versus 0.63 g/d in the control. Specific growth rates (SGR) also reflected this trend, with values of 2.29 %/d and 2.24 %/d for the 1g/kg - and 2g/kg treatment groups respectively compared to 1.07 %/d for the control. Overall, zinc chelate of glycine significantly (*P* < 0.05) enhances growth performance and survival in hybrid grouper in 30 days, particularly at the 1g/kg (0.1%) concentration.

**Fig. 4:**
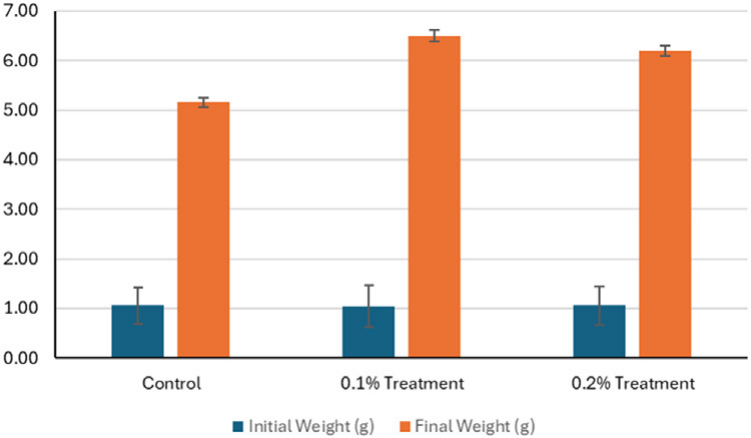
The initial weight (g) and final weight (g) of the whiteleg shrimp in control and treatment after 6 weeks of trials. The control group feed was prepared without the inclusion of zinc chelate of glycine. As for the treatment 0.1, feed was top-coated with zinc chelate of glycine at a concentration of 1 g/kg of feed and for the treatment 0.2, feed was top-coated with zinc chelate of glycine at a concentration of 2 g/kg of feed. Error bars represents the standard deviation of the replicates.

**Fig. 5:**
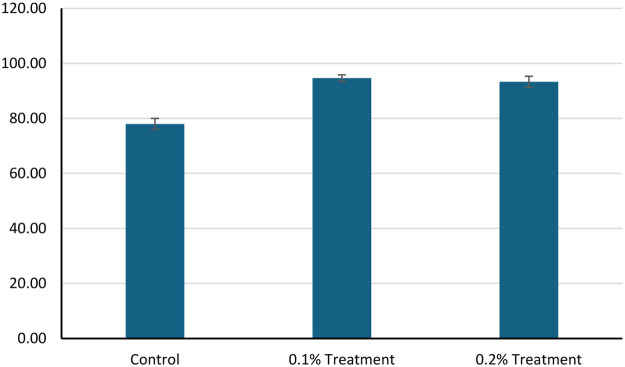
The survival (%) of the whiteleg shrimp in control and treatment after 6 weeks of trials. The control group feed was prepared without the inclusion of zinc chelate of glycine. As for the treatment 0.1, feed was top-coated with zinc chelate of glycine at a concentration of 1 g/kg of feed and for the treatment 0.2, feed was top-coated with zinc chelate of glycine at a concentration of 2 g/kg of feed. Error bars represent the standard deviation of the replicates.

The similar pattern of the data was observed in the shrimp fed with the 0.1% and 0.2% zinc chelate of glycine. The initial weights of whiteleg shrimp were comparable across groups, with values of 1.05 g for control and 1.04 g for 0.1% and 0.2% zinc chelate of glycine group. By the end of the study, final weights increased significantly, reaching 5.15 g in the control group, 6.50 g for the 0.1% zinc chelate of glycine fed group, and 6.20 g for the 0.2% zinc chelate of glycine fed group (Fig. 4). Survival rates improved significantly (*P* < 0.05) in the zinc chelate of glycine fed groups, with 94.67% survival at 0.1% and 93.33% at 0.2%, compared to 78.00% for the control (Fig. 5). The weight gain (g) was also higher in the zinc chelate of glycine groups, with 5.46 g for the 0.1% treatment and 5.15 g for the 0.2% treatment, compared to 4.10 g for the control. Average daily gain (ADG) reflected similar trends, showing values of 0.14 g/d for the control, 0.18 g/d for the 0.1% zinc chelate of glycine fed shrimp, and 0.17 g/d for the 0.2% zinc chelate of glycine fed shrimp. Specific growth rates (SGR) were higher in the zinc chelate of glycine fed groups, with rates of 6.11 %/d and 5.92 %/d for the 0.1% and 0.2% treatments, respectively, compared to 5.30 %/d for the control in 6 weeks of treatment.

## 4 Experimental Design, Materials and Methods

### 4.1 Feed preparation

Commercial diet was used for the feeding trial while for the control group, feed was prepared without the inclusion of Aqua Ectogon-284 (zinc chelate of glycine) For Treatment 1, feed was top-coated with zinc chelate of glycine at a concentration of 1 g/kg of feed. For Treatment 2, feed was top coated with zinc chelate of glycine at a concentration of 2 g/kg of feed. Zinc chelate of glycine was mixed with 2% of water. The mixture was evenly sprayed onto the commercial diet to ensure homogenized distribution of the supplement and mixed for 15 minutes.

### 4.2 Experimental design and animal husbandry

#### 4.2.1 Hybrid grouper trial

Commercial diet (Star feed, CP Group) with a composition of. 43% protein and 6% lipid were used for the feeding trial. For the control group, feed was prepared without the inclusion of zinc chelate of glycine. For Treatment 1, feed was top coated with zinc chelate of glycine at a concentration of 1 g/kg of feed. For Treatment 2, feed was top coated with zinc chelate of glycine at a concentration of 2 g/kg of feed. Zinc chelate of glycine was mixed with 2% of water. The mixture was evenly sprayed onto the commercial diet to ensure homogenized distribution of the supplement and mixed for 15 minutes.

**Fig. 6:**
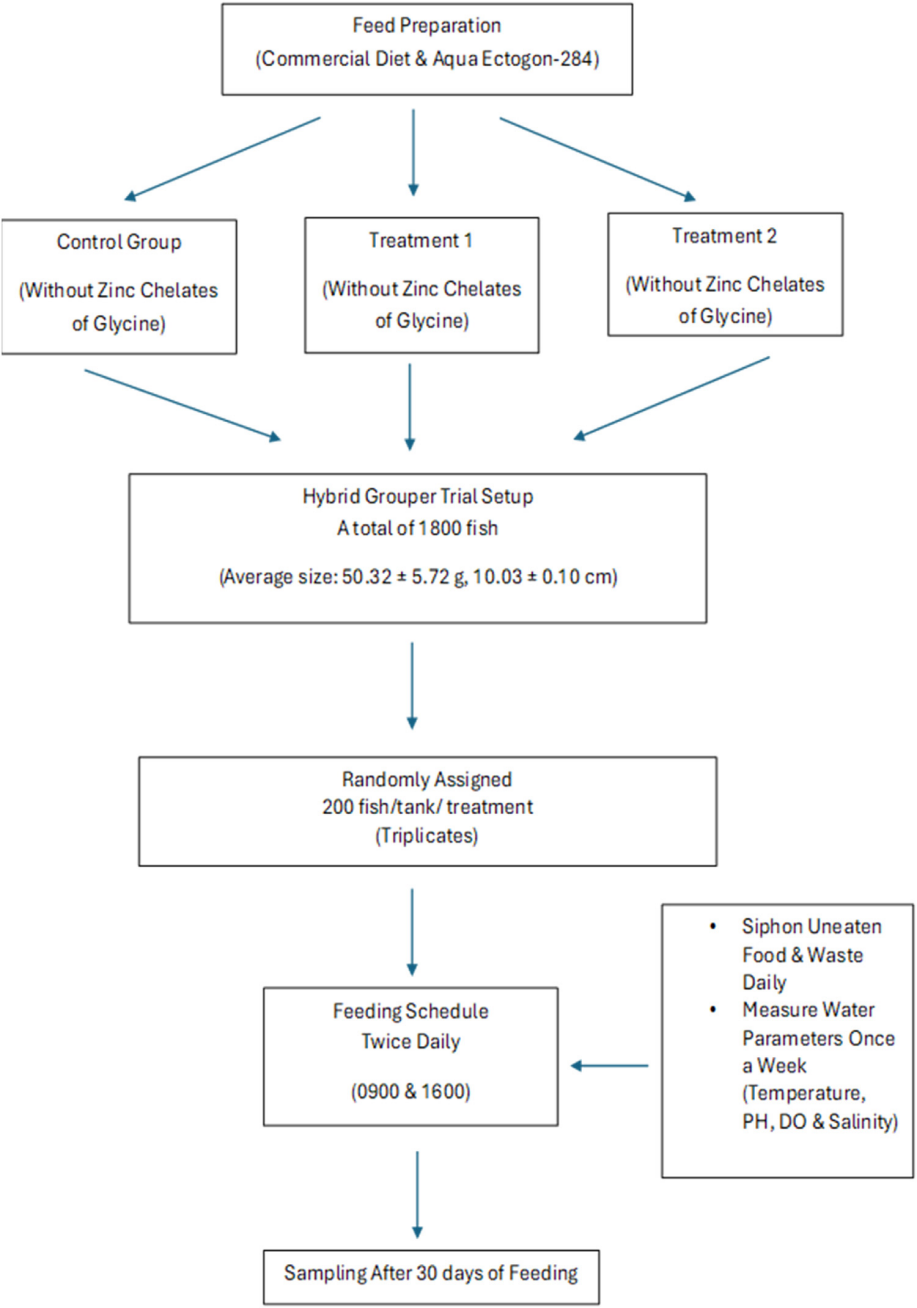
Experimental flowchart of hybrid grouper (*Epinephelus fuscoguttatus* ♀ × *E. lanceolatus* ♂) trial. Experiments for each group were conducted in triplicate. The dataset for this experiment can be obtained at https://data.mendeley.com/datasets/8sc8n33tmh/1.

The hybrid grouper (*Epinephelus fuscoguttatus* ♀ × *E. lanceolatus* ♂) was obtained from a local farm nursery, and the feeding trial was carried out there. A total of 1800 fish, with an average size of 50.32 ± 5.72 g and 10.03 ± 0.10cm, were weighed and distributed into each concrete tank (3m x 2m x 1m). Each treatment group was randomly assigned in triplicate, with 200 fish per treatment. Fish were fed twice daily at 0900 and 1600 until apparent satiation, with each feeding session lasting 30 minutes to ensure all fish were adequately fed. Each day, any uneaten food was siphoned out. Temperature, pH, DO, and salinity were measured once a week using a Horiba U50 device. During the feeding trial, temperatures ranged from 28-29.1°C, pH was between 7.9-8.2, salinity was around 30-32 ppt, and DO levels were maintained above 6.3 ppm. After 30 days of feeding, all fish were harvested and measured for weight and length. Fig. 6 outlines the flowchart of the experimental design for hybrid grouper trials.

#### 4.2.2 Whiteleg shrimp trial

Commercial diet, De heus Ruby SP (32-34% protein) with a composition of Min. 34% protein and 5% lipid was used for the feeding trial. For the control group, feed was prepared without the inclusion of zinc chelate of glycine. For Treatment 1, feed was top coated with zinc chelate of glycine at a concentration of 1 g/kg of feed. For Treatment 2, feed was top coated with zinc chelate of glycine at a concentration of 2 g/kg of feed. Zinc chelate of glycine was mixed with 2% of water. The mixture was evenly sprayed onto the commercial diet to ensure homogenized distribution of the supplement and mixed for 15 minutes.

**Fig. 7:**
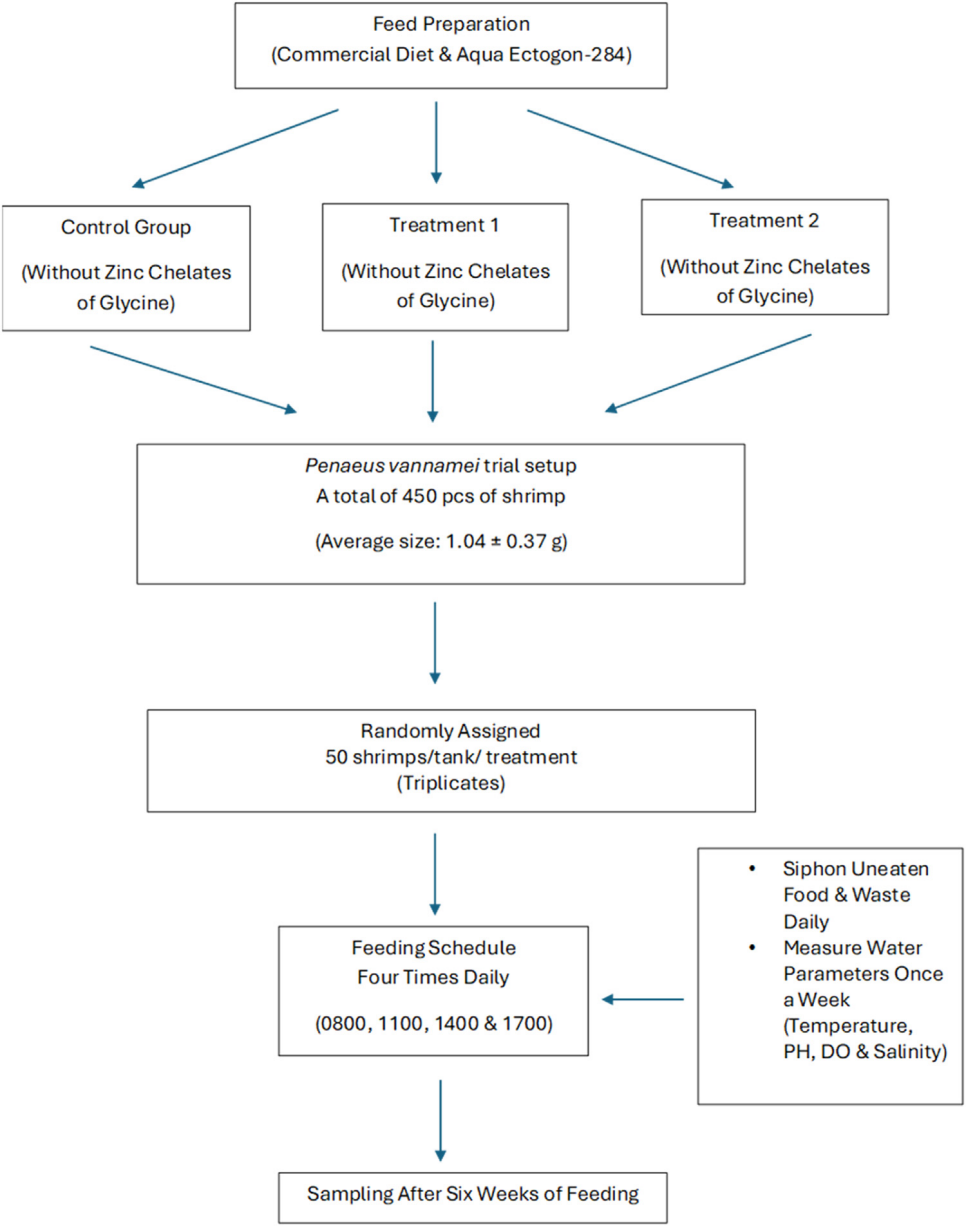
Experimental flowchart of whiteleg shrimp, *Penaeus vannamei* trial. Experiments for each group were conducted in triplicate. The dataset for this experiment can be obtained at https://data.mendeley.com/datasets/8sc8n33tmh/1.

The whiteleg shrimp, *P. vannamei* was obtained from a local farm nursery, and the feeding trial was carried out there. A total of 450 pieces of shrimp, with an average size of 1.04 ± 0.37 g, were weighed and distributed into each concrete tank (1.5m x 2m x 1.5m). Each treatment group was randomly assigned in triplicate, with 50 shrimp per treatment. Whiteleg shrimp were fed 4 times daily at 0800, 1100,1400 and 1700 at a rate of 5% body weight. Each day, any uneaten food was siphoned out. Temperature, pH, DO, and salinity were measured once a week using a Horiba U50 device. During the feeding trial, temperatures ranged from 28-29.1°C, pH was between 8-8.2, salinity was around 30-32 ppt, and DO levels were maintained above 6.1 ppm. After 6 weeks of feeding, all shrimps were harvested and measured for weight and length. Fig. 7 outlines the flowchart of the experimental design for whiteleg shrimp trials.

### 4.3 Survival & growth performance

At the end of the feeding trial, the survival rate (%), final weight (g), final length (cm), weight gain (g) and specific growth rate (SGR, %) were determined based on the following equations:

Survival(%)=(finalamountofanimal/initialamountofanimal)X100%

WeightGain(g)=finalweight(g)−initialweight(g)

FinalLength(cm)=finallength(cm)−initiallength(cm)

$$\text{Specific}\;\text{Growth}\;\text{rate}\;\left(\%/\text{day}\right)\;=\frac{\ln\left(final\;weight\;\left(g\right)\right)-\ln\left(initial\;weight\;\left(g\right)\right)}{Feeding\;Period\;\left(days\right)}X\;100\%$$

### 4.4 Statistical analyses

All data of final weight, final length, weight gain, and survival rates across the different treatment groups (control, 0.1% treatment, 0.2% treatment) were subjected to one-way ANOVA, followed by Tukey's post-hoc test, after prior confirmation of normality and homoscedasticity. All data are presented as mean ± standard deviation and statistical analyses were performed using SPSS version 28.x.

## Limitations

Not applicable.

## Ethics Statement

The authors confirm that all experiments conducted in this study comply with the ARRIVE guidelines and were performed in accordance with the U.K. Animals (Scientific Procedures) Act 1986, the associated guidelines, the EU Directive 2010/63/EU on the protection of animals used for scientific purposes, and the National Institutes of Health Guide for the Care and Use of Laboratory Animals (NIH Publications No. 8023, revised 1978).

## CRediT Author Statement

**Wee Wen Chen & Sarmila Muthukrishnan:** Conceptualization, Data collection and Drafting the manuscript; **Donny Tey Kai Hee:** Review and editing; **Sarmila Muthukrishnan:** Data analysis, Formatting, reviewing and submission.

## Data Availability

Mendeley DataZinc chelates of glycine as a growth and survival enhancer in hybrid grouper and whiteleg shrimp (Original data).
